# Reducing Agricultural Land Use Through Plant-Based Diets: A Case Study of Romania †

**DOI:** 10.3390/nu17010175

**Published:** 2025-01-02

**Authors:** Ioana Mihaela Balan, Teodor Ioan Trasca

**Affiliations:** 1 University of Life Sciences “King Mihai I”, 300645 Timisoara, Romania; 2 University of Agronomic Sciences and Veterinary Medicine of Bucharest, 011464 Bucharest, Romania

**Keywords:** plant-based diet, agricultural land use, sustainability, protein source replacement, Sustainable Development Goals (SDGs)

## Abstract

Background/Objectives: Agricultural systems face increasing global pressure to address sustainability challenges, particularly regarding land use and environmental protection. In Romania, where traditional diets are heavily dependent on animal-based products, optimizing land use is critical. This study investigates the potential of plant-based diets to reduce agricultural land use, examining scenarios of partial and complete replacement of animal protein with plant protein sources (soy, peas, and potatoes). Methods: The research modeled three dietary transition scenarios—replacing 33%, 50%, and 100% of animal protein with plant-based protein—using data from the Romanian National Institute of Statistics, the FAO, and international sources. Land use was calculated for each scenario using formulas that take into account protein content and land use intensity for animal and plant protein sources. The simulations quantify the reduction in agricultural land use at the per capita and national levels. Results: The study reveals significant land-saving potential across all scenarios. At the national level, land use reductions ranged from 84,020 hectares (33% replacement) to 1,067,443 hectares (100% replacement). High-impact products such as beef and dairy continue to dominate land use, even in partial replacement scenarios. Conversely, replacing pork and chicken proteins shows substantial savings. The findings highlight the inefficiency of animal-based protein production and the ecological benefits of transitioning to plant-based diets. Conclusions: A dietary shift towards plant-based protein in Romania could achieve significant reductions in agricultural land use, contributing to the Sustainable Development Goals. This transition not only supports environmental conservation and resource optimization, but also provides public health benefits by reducing consumption of red and processed meat. These results provide a basis for policies to promote sustainable and nutritionally balanced food systems

## 1. Introduction

This research was originally presented at The 4th International Electronic Conference on Nutrients, where the abstract was published as part of the conference materials [1].

In recent decades, agriculture has faced increasing pressure from global demands for sustainability and environmental protection. As the world’s population grows [2,3], the need for agricultural land for food production intensifies, and current agricultural practices are increasingly challenged from the perspective of the impact on natural resources, such as water, land, and biodiversity. In this context, plant-based diets have become a central solution in global discussions about sustainability. Adopting such a diet, which requires fewer resources for protein production, could bring significant benefits in terms of land use, environmental protection, and climate change mitigation.

Romania, a country with predominantly traditional agriculture, has considerable potential to adopt such sustainable methods, especially considering the agricultural sector represents an important part of the national economy [4,5]. Currently, a large part of the agricultural land in Romania is dedicated to raising animals and growing fodder for them, which contributes to an intensive and inefficient use of land. International studies indicate that the production of protein from meat, especially from beef, requires up to ten times more land than the production of protein from cereals or legumes [6,7,8]. This makes plant-based diets much more efficient from a land use perspective [9].

International organizations, including the FAO and the Lancet Commission, highlight the urgency of adopting plant-based diets to conserve resources, protect biodiversity, and reduce greenhouse gas emissions [10,11,12,13]. Such diets are less resource-intensive and offer a viable path to optimizing agricultural land use.

For Romania, aligning with these international trends represents an opportunity to reduce the agricultural footprint and contribute to national and global sustainability goals. Although the country has a long history of high consumption of animal products, there is a growing interest among the public and policymakers for solutions to address environmental challenges [14]. Studies from other European countries show that a transition to plant-based diets could reduce agricultural land use by up to 75% [15]. These data are also relevant for Romania, which faces similar pressures in terms of land use and the need to preserve natural ecosystems.

International research on the impact of plant-based diets on land use is well documented and supported by clear evidence. FAO studies have shown that diets high in meat, especially beef, have a disproportionate impact on agricultural land. For every gram of protein produced from beef, it takes much more land than to produce the same amount of grams of protein from legumes or cereals. Similarly, the Lancet Commission emphasized the importance of adopting a healthier, plant-based diet as part of a global effort to reduce the impact of agriculture on the environment [12,13].

However, research in Romania on this subject remains limited. While there are some studies exploring the impact of intensive agriculture on the land and environment, few focus on the potential for transitioning to plant-based diets. This knowledge gap is particularly important, considering Romania’s agricultural specifics and the importance of agriculture for its economy. The present study aims to fill this gap, providing a detailed analysis of the impact that such a dietary change would have on the use of agricultural land in Romania.

We also investigated how adopting a plant-based diet can help reduce agricultural land use in Romania. The main goal is to quantify the land savings that could be achieved by replacing animal protein sources with plant sources such as legumes and cereals. The study is based on data provided by government and international sources and examines the efficiency of land use in protein production. The models used in this study are inspired by previous research conducted for the transition from animal-based to plant-based food production in other countries, which were never adapted to the Romanian context [16,17,18].

The research also explores the impact of such a dietary transition on the long-term sustainability of Romanian agriculture. It is estimated that a transition to a predominantly plant-based diet could reduce the use of agricultural land by up to 75%, a result that would have significant implications not only for the land used but also for the protection of natural resources and biodiversity. In addition, the transition to more sustainable diets could help reduce emissions from agriculture and improve public health [15].

Romania has a vast agricultural area, occupying almost 60% of the national territory, and is one of the largest producers of cereals, such as corn and wheat, in the European Union [19]. However, much of this land is devoted to livestock and fodder cultivation, leading to an intensive and sometimes inefficient use of resources.

The fragmentation of agricultural land, the predominance of small farms, and the lack of modern technology contribute to low productivity in many rural areas. Romanian agriculture is also vulnerable to climate change, due to high dependence on weather conditions and aging irrigation infrastructure. Animal husbandry plays a central role, but meat and dairy production exerts significant pressure on agricultural land [20].

In this context, the adoption of a plant-based diet could have a significant impact on Romanian agriculture, freeing up land used for livestock and fodder, contributing to the optimization of the use of agricultural resources. The transition to plant-based food production, such as legumes and cereals, could not only reduce the pressure on the land but also support Romania’s sustainability goals, which are aligned with European strategies [12,21]. Such a change could stimulate the modernization of the agricultural sector, encouraging the implementation of more efficient and sustainable practices, as well as the adoption of new technologies.

Therefore, a plant-based diet would not only contribute to reducing the use of agricultural land but would also create opportunities for increased sustainability in agriculture, providing solutions for the current challenges facing Romania [19].

The implementation of a dietary transition of this type could have substantial benefits for Romania. In addition to reducing pressure on agricultural land, this change could support the adoption of more sustainable and greener agricultural practices. In addition, the release of significant areas of agricultural land would allow their use for reforestation or other activities aimed at supporting the conservation of natural ecosystems [22,23,24].

From a public policy perspective, the study provides a possible basis for formulating sustainable strategies in the field of food and agriculture. As Romania faces major challenges in terms of natural resource management and environmental protection, adopting a plant-based diet may be a viable solution to reduce the negative impact of agriculture on the environment. Using the data provided by this study, policymakers can develop educational programs and awareness campaigns that encourage the public to adopt healthier and more environmentally friendly eating habits.

Therefore, this research demonstrates that a transition to a plant-based diet in Romania has the potential to bring significant ecological, economic, and health benefits. This change could serve as a model applicable not only at the national level, but also in other countries facing similar challenges related to agricultural land use and food sustainability.

## 2. Materials and Methods

### 2.1. Data and Sources

To carry out this study on reducing agricultural land use through plant-based diets in Romania, we collected and analyzed external data from relevant international and national institutions. Statistical data comes from Our World in Data (Oxford, Great Britain), the FAO, and the Romanian National Institute of Statistics (INSEE). In parallel, we analyzed the scientific literature from major international databases such as Web of Science, Scopus, PubMed, ScienceDirect, and gray literature. The literature selection process involved the use of keywords such as “land use for animal products”, “protein land footprint”, “plant-based protein sustainability”, and “animal vs. plant protein environmental impact”. We identified 36 relevant bibliographic references, 7 of which are from the authors of the present study, addressing the consumption of animal products, their impact on agricultural land use, and recommended dietary patterns, providing a diversified and up-to-date basis for analysis. The selection of these sources was made based on direct relevance to land use comparisons between animal and plant protein sources but also on the accessibility and authority of these data.

To assess the impact of a plant-based diet on agricultural land use in Romania, this study adopted a modeling-based approach, combining data from governmental and international sources with models adapted to Romanian agricultural specificity. The models were built to quantify the potential agricultural land savings resulting from the transition to a plant-based diet, making comparisons between the agricultural land required to produce animal-based products versus plant-based products. These models have been adjusted to reflect average agricultural production conditions in Romania, using data provided by the FAO and Our World in Data.

In this research, we chose to focus on the protein content of foods as a central element of comparison between animal and vegetable products. This decision was made based on the fundamental importance of protein in the diet and its essential role in ensuring balanced nutrition [25]. Proteins are essential macronutrients required for many vital functions, such as tissue repair, enzyme and hormone production, and muscle mass maintenance. Comparing protein content is also essential to assessing the ecological impact of different food sources, given that animal products are particularly valued for their high protein content.

Replacing animal protein with plant protein requires careful consideration to ensure nutritional equivalence and to identify the most efficient plant sources in terms of resource use. Protein content is a suitable indicator for assessing the sustainability of a diet, as it allows direct comparison of different protein sources according to the land required for their production [25,26]. Although there are other important nutrients in food (such as carbohydrates and fats), protein was chosen as the main comparator because it provides a concrete and critical measure of nutritional intake and agricultural land use efficiency. Thus, focusing on protein allows us to assess the ecological impact of changing protein sources, providing a solid basis for understanding how a transition to a plant-based diet can contribute to saving agricultural resources without compromising the nutritional needs of the population [27,28,29].

### 2.2. Applied Formulas

In this research, we decided to analyze the impact on land use by simulating the gradual replacement of animal protein with vegetable protein. To provide a clear and detailed picture of the land economy, we set three replacement scenarios: 33%, 50%, and 100%. These rates were chosen to reflect different levels of dietary transition and to progressively analyze how the adoption of plant-based diets could reduce pressure on agricultural land.

(a) *The 33%-replacement scenario.* This rate was selected because it represents a moderate reduction and is easier to achieve in practice. Replacing a third of animal protein with plant-based protein can be an affordable first step for the population, providing a balance between nutritional intake and reducing the impact on agricultural land. This scenario provides a realistic perspective on a partial transition to more sustainable diets.
(b) *The 50%-replacement scenario.* This scenario represents a significant reduction in animal protein consumption and marks an intermediate transition to a largely plant-based diet. The 50% rate allows for a detailed assessment of the effects of a more ambitious change in agricultural land use, providing a picture of how such a regime can influence the sustainability of food systems.
(c) *100%-replacement scenario.* This represents an extreme scenario where all animal proteins are replaced by vegetable proteins. Although such a complete change may seem less realistic to some consumers, it is important to evaluate the maximum possible impact of switching to a completely plant-based diet. This scenario highlights the maximum land savings that could be achieved by completely eliminating animal products from the diet.

Through these three replacement scenarios, our analysis provides insight into how different degrees of dietary change can reduce agricultural land use. These scenarios reflect both gradual changes, which might be easier to implement among the population, and a complete change, to show the maximum potential for sustainability.

To assess the impact on land use following the replacement of animal proteins with vegetable proteins, we used the following three formulas.

The first calculates the agricultural land use for currently consumed animal proteins (Land Use initial), the second calculates the use of land for vegetable proteins that replace animal proteins (Land Use with replaced proteins), and the third (Land Use final) reveals the difference between the first two, related to the three scenarios of replacing animal protein with vegetable protein (33%, 50%, and 100%).

*Formula for Land Use initial:*

(1) $${LU}_{initial}=\left(\frac{C\cdot P}{10}\;\right)\cdot {LU}_{animal}$$

*C*—Annual per capita consumption of the product of animal origin (kg)
*P*—Percentages of protein per 100 g of food of animal origin (%)
*LU_animal_*—Land use per 100 g of animal protein (m^2^).

*Formula for Land Use with replaced proteins:*

(2) $${LU}_{replaced,x}=\left(\frac{C\cdot P}{10}\;\right)\cdot R_{x}\cdot \sum_{i=1}^{n}(w_{i}\;\cdot {LU}_{vegetal,i})$$

*C*—Annual per capita consumption of the product of animal origin (kg)
*P*—Amount of protein per 100 g of food of animal origin (g)
*R_x_*—Percentage of animal protein replaced
*n*—Number of plant-based protein sources
*i*—Order number of the plant-based protein source in the replacement group
*W_i_*—Proportion of plant-based protein that contributes to the replacement
*LU_vegetal,i_*—Land use per 100 g of protein from plant-based source *i* (m²).

*Formula for Land Use final:*

(3) $${LU}_{final}={LU}_{initial}-{LU}_{replaced}$$

This difference reflects the economy of agricultural land following the substitution of animal proteins for vegetable proteins for each reduction scenario (33%, 50%, 100%).

### 2.3. Simulations and Scenarios

In our research, the structure of replacing animal proteins with vegetable proteins played a central role. We chose three essential plant sources—soy, peas, and potatoes—to simulate different replacement rates, taking into account both the food preferences of the Romanian population and the efficiency of these foods in terms of agricultural land use [30,31,32].

This combination was used to analyze the impact on the agricultural areas needed to support the Romanian diet, which is currently heavily dependent on meat and dairy products. By using 50% soybeans, 30% peas, and 20% potatoes, we evaluated the ability of these foods to meet the protein needs of the population, but with a low ecological impact.

This approach also allows us to model different scenarios of partial or total replacement of animal protein and see how each scenario affects the use of land resources. Thus, our research not only proposes a sustainable alternative to the traditional diet, but also provides concrete data to support a viable transition to a greener food system.

We ran simulations to estimate the effects of different scenarios for reducing the consumption of animal products. The simulations included scenarios of a gradual transition to an exclusively plant-based diet, that is, from reducing the consumption of animal products to a full transition to a vegan diet.

The calculation was applied for each type of food of animal origin, in three replacement stages, related to the three scenarios:

(a) 33% of animal proteins were replaced by plant-based proteins;
(b) 50% of animal proteins were replaced by plant-based proteins; and
(c) 100% of animal proteins were replaced by plant-based protein.

These scenarios were used to quantify the impact of dietary transitions on agricultural land use and to explore how the freed-up land could be reused for ecological or agricultural purposes. This modeling approach highlights the land-saving potential of plant-based diets at different levels of reduction.

### 2.4. Analysis of the Results

The data generated from these simulations were analyzed to emphasize the land-saving potential and its implications for the sustainability of Romanian agriculture. The analysis also considered how reallocated land could be used for reforestation or ecosystem restoration, offering additional ecological benefits.

The methodology of this study provides a basis for understanding the impact of a major dietary change on agricultural land in Romania and can serve as a reference for future research in the field of agricultural sustainability.

## 3. Results

In Romania, products of animal origin, especially meat and milk, together with their derivatives, such as sausages and dairy products, constitute a significant part of the daily diet of the population [20,29]. The high consumption of meat—especially pork, chicken, and beef, but also dairy products—reflects the traditional and cultural preferences of Romanians, who have a long history of eating foods rich in animal proteins. These products are associated with culinary habits that emphasize satiety and their constant presence in the main meals of the day [25,26].

However, the negative impact on the environment and natural resources caused by the intensive use of land to produce these foods led us to explore more sustainable alternatives based on plant sources [33,34,35]. Building on this context, the research simulated the partial replacement of animal proteins with plant-based alternatives to quantify potential agricultural land savings and support a transition toward sustainable dietary practices.

We opted for the following plant sources in our simulations:

- *Soy—50% of the replacement*. We chose soy because of its high protein profile and health benefits. Soy is a complete protein source, containing all the essential amino acids, making it an excellent alternative to animal protein. In addition, soy consumption is associated with cardiovascular health benefits and reduced risk of chronic disease [36,37]. Compared to beans, soybeans are more efficient in providing quality protein with low land impact.
- *Peas—30% of the replacement.* Peas are a highly valued vegetable in Romania and are frequently consumed, both fresh and canned. They are rich in proteins and fibers and easy to integrate into various traditional culinary preparations. Peas also provide high-quality protein, although their content of essential amino acids is lower than that of soy, which is why we allocated a lower percentage to this source [38,39].
- *Potatoes—20% of the replacement*. Potatoes are a staple food in the Romanian diet and one of the most consumed vegetables [40,41]. Although they are not very rich in protein, potatoes complete the caloric intake and are extremely versatile in Romanian cuisine [42,43]. Therefore, we thought it appropriate to include potatoes in our simulation given their popularity and relatively low impact on farmland.

**Figure 1:**
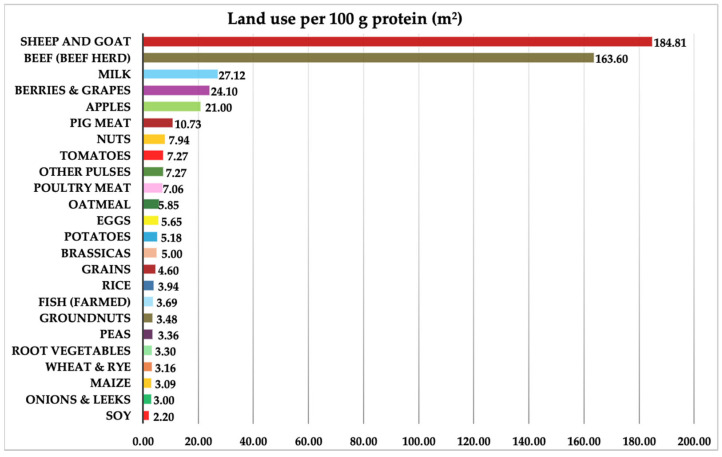
Land use per 100 g protein for various food categories (m²). Source: Original by authors after [45].

Through this replacement structure, we wanted to maintain a balance between quality proteins and the foods preferred by Romanians, while also analyzing the impact on the use of agricultural land. Of course, this replacement structure is only created for the purpose of this study, as an example. In practice, the replacement structure can be modified according to consumer preferences, both with the plant-based protein sources shown in Figure 1, as well as many others not included in the study because they are not specific to the cultivation conditions, nor are they traditionally cultivated in Romania (e.g., cassava) [44].

Comparing land use for the production of 100 g of protein, we see major differences between animal and plant-based products. Based on the collected data, we analyzed the annual per capita consumption of animal products, which is composed of meat, fish and seafood, and milk. The meat and milk groups also include equivalent processed products (sausages and dairy products). We collected data on the protein content presented in Table 1, which provides a detailed look at the protein content for various animal products, expressed in grams of protein per 100 g of product. Data are extracted from an FAO reference source and reflect average protein values for types of meat, fish and seafood, eggs, and milk [22].

Table 2 presents the annual per capita consumption of animal products in Romania in 2021, including both meat and its equivalents in the form of meat preparations. Also, milk data includes the consumption of equivalent dairy products. The information reported by Our World in Data is related to per capita consumption and provides a clear picture of the food preferences of the Romanian population [23].

Pork and chicken are predominant in the diet, reflecting a culinary tradition that favors these types of meat. The raising and exploitation of pigs in Romania is traditional, and the high consumption of pork meat is associated with various traditional events [29]. At the same time, the consumption of dairy products is particularly high, indicating a significant dependence on milk and its derivatives. These data reflect the traditional preferences of Romanians for pork and dairy products, which have a major presence in the daily diet [46,47]. Fish and beef consumption remain low, in line with trends seen in Eastern Europe [48].

We modeled the three land use scenarios for meat, milk, and egg protein production compared to plant-based sources, such as legumes and cereals, and applied Formulas (1)–(3). The results for the three scenarios are reproduced below.

### 3.1. Scenario 1—33% of Animal Protein Replaced by Plant-Based Protein

In the first scenario, we simulate the impact of a 33% reduction in animal protein consumption, replaced by vegetable protein sources (soy 50% replacement, peas 30% replacement, and potatoes 20% replacement). Table 3 shows the initial land use for each category of animal product, as well as the decrease in land use after applying this 33% partial replacement of animal protein with vegetable protein, according to Formulas (1)–(3).

In this first scenario, the impact on land use varies based on the share of each food in per capita consumption. Milk, with a very high consumption in Romania (599.32 kg per capita), and beef, despite lower consumption, have significant ecological footprints due to the extensive land required for their production. Products with lower consumption, such as eggs and fish, contribute less to land savings, while reducing high-impact products like milk yields greater benefits.

The 33% reduction in animal protein consumption, related to total consumption, results in a modest but meaningful decrease in land use: from 8164.59 m² per capita to 8108.35 m², saving 56.24 m² per capita. Beef remains impactful, with land use decreasing slightly from 1528.39 m² to 1518.70 m². Milk also has a significant reduction, from 5363.91 m² to 5158.72 m², despite its continued dietary importance.

In contrast, chicken and pork show more noticeable reductions due to higher consumption. Land use for chicken decreases from 267.96 m² to 228.55 m² and pork from 435.50 m² to 393.39 m², highlighting the potential for greater impact even with partial changes in consumption

Eggs and fish, despite their relatively low consumption in Romania, contribute to total land use, and under the 33% replacement scenario, reducing their consumption results in land savings of approximately 14.25 m² and 9.15 m², respectively.

This scenario demonstrates that a partial reduction in animal protein consumption can generate significant agricultural land savings, especially for the pork and chicken categories. However, products such as milk and beef continue to dominate land use, suggesting that a more drastic change would be needed to see significant reductions in the ecological footprint. This scenario sets the stage for the following simulations, where we explore the impact of 50% and 100% reductions.

### 3.2. Scenario 2—50% of Animal Protein Replaced by Plant-Origin Protein

In this scenario, we simulate a more substantial reduction by replacing 50% of the animal protein consumed with plant sources. Table 4 shows land use before and after replacement, highlighting the economy of agricultural land for each product category, according to Formulas (1)–(3).

In Scenario 2, a 50% replacement of animal protein with plant-based protein results in a substantial reduction in land use: from 8164.59 m² to 7941.34 m² per capita, saving 223.25 m². This scenario highlights the growing impact on land resources as the replacement percentage increases.

Milk remains the dominant contributor to land use due to its high consumption, with savings of 310.90 m² as land use decreases from 5363.91 m² to 5053.01 m². Beef, despite its high ecological footprint per unit, shows only a slight reduction from 1528.39 m² to 1513.71 m², owing to its relatively low consumption of 5.05 kg per capita. Pork and chicken exhibit the most significant reductions: pork land use decreases from 435.50 m² to 371.69 m² and chicken from 267.96 m² to 208.26 m², reflecting the considerable benefits of shifting to plant-based sources.

Eggs and fish, while consuming less land than pork and chicken, also contribute to land savings, with a reduction of 21.60 m² for eggs and 13.86 m² for fish and seafood.

This scenario clearly illustrates that adopting a 50% reduction in animal protein consumption and replacing it with vegetable protein can generate considerable land savings. However, products such as beef and milk continue to be large consumers of land, even after such a reduction. On the other hand, pork and chicken show more substantial savings, suggesting that through a balanced dietary change, more favorable results can be achieved in terms of agricultural resource management. This scenario brings us closer to understanding the full impact of a wider dietary transition, which we will explore in detail in the next scenario, where we look at the complete replacement of animal protein.

### 3.3. Scenario 3—100% of Animal Proteins Replaced by Plant-Origin Proteins

In this scenario, all animal proteins are completely replaced by plant sources (soy, peas, and potatoes). This radical transition requires a careful analysis of how each category of animal products contributes differently to land use, both before and after replacement. The transition affects food categories differently due to their specificities in per capita consumption and impact on land resources.

This scenario reflects a complete transition to plant-based protein sources, where all animal proteins are eliminated from consumption. After replacement, final land use (LU_final_) represents the resulting land use calculated according to Formulas (1)–(3) (Table 5).

In Scenario 3, the complete replacement of animal proteins with plant sources leads to a substantial reduction in agricultural land use, from 8164.59 m² to 7450.14 m² per capita, representing a total saving of 714.45 m². The impact of this transition varies depending on the food categories, in relation to their consumption levels and ecological footprint. Milk, the most consumed animal product in Romania (599.32 kg per capita annually), continues to dominate land use, with a reduction from 5363.91 m² to 4742.11 m². Although saving 621.80 m², milk remains a major contributor due to its high consumption. Pork, another important product, with a consumption of 36.9 kg per capita, records a decrease in land use from 435.50 m² to 307.89 m², saving 127.61 m². Chicken, consumed at 22.21 kg per capita, also shows significant reductions, with land use decreasing from 267.96 m² to 148.55 m². Beef, although consumed in smaller quantities (5.05 kg per capita), remains highly inefficient, with land use only decreasing from 1528.39 m² to 1499.02 m². Sheep and goats, despite a low consumption (2.8 kg per capita), also have a large ecological footprint, with land use decreasing from 724.47 m² to 712.14 m². These categories highlight the disproportionate impact of products with high environmental demands per unit.

Smaller categories, including fish and seafood (8.09 kg per capita) and eggs (12.84 kg per capita) contribute modestly to land savings. Land use for fish decreases from 32.52 m² to 4.80 m² and, for eggs, from 77.63 m² to 34.44 m². The “other meats” category, which includes products such as turkey and rabbit, reduces land use from 2.16 m² to 1.20 m².

Thus, Scenario 3 demonstrates the potential for significant environmental benefits from a full transition to a plant-based diet. While high-consumption categories such as milk and pork continue to dominate land use even after replacement, lower-consumption categories such as fish, eggs, and other meats offer additional cumulative savings, highlighting the importance of a holistic approach to reducing pressure on agricultural land.

### 3.4. The Impact of Food Transition Scenarios on the Use of Agricultural Land in Romania

This research applies a model to evaluate the impact that reducing the consumption of animal protein and replacing it with plant-based protein would have on the use of agricultural land in Romania. Focusing on the data on the adult resident population of Romania from 2021—the reporting year of animal food consumption—which was 14,940,830 people, we apply the calculations to understand the impact at the national level [48] (Table 6).

In this context, we consider three reduction scenarios: 33%, 50%, and 100%. Scenario 1 assumes a 33% replacement of animal protein, with an estimated impact of reducing the agricultural land needed per capita, extrapolated to the entire adult resident population in Romania, providing an understanding of the reduction of agricultural land used. Scenario 2 increases this replacement to 50%, providing insight into greater land-saving potential. Scenario 3 explores the impact of a full (100%) transition to plant-based proteins, illustrating the most optimistic scenario for reducing agricultural land use.

Each of these scenarios is calculated by multiplying per capita land use by the total adult population, thus providing concrete data on total land savings at the national level. The obtained results underline the significant potential of reducing Romania’s ecological footprint through dietary changes, providing a solid foundation for sustainable natural and food resource management policies.

**Figure 2:**
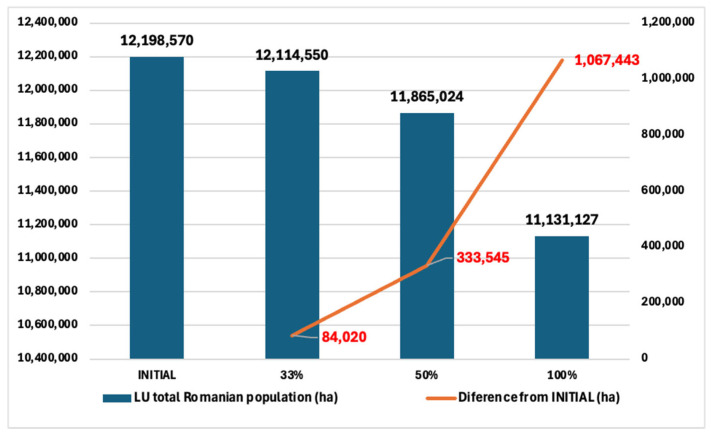
Impact of protein replacement scenarios on total land use in Romania. Source: Original by authors.

Figure 2 shows the impact of the simulations to reduce the consumption of animal products on the use of agricultural land in Romania, expressed in hectares (ha).

We note that initially, for 14,940,830 adult Romanian residents, the total land area used is 12,198,570 ha. With the reduction of 33%, the area drops by 84,020 ha, which represents more than the utilized agricultural area (UAA) of Ilfov County (61,987.41 ha) [49].

With the reduction of 50%, the area drops further to 11,865,024 ha, with an additional decrease of 249,525 ha from the previous scenario, totaling a savings of 333,545 ha from the baseline, which represents almost all the UAA of the mid-sized county in Romania, Galati County (332,030.23 ha) [49].

**Figure 3:**
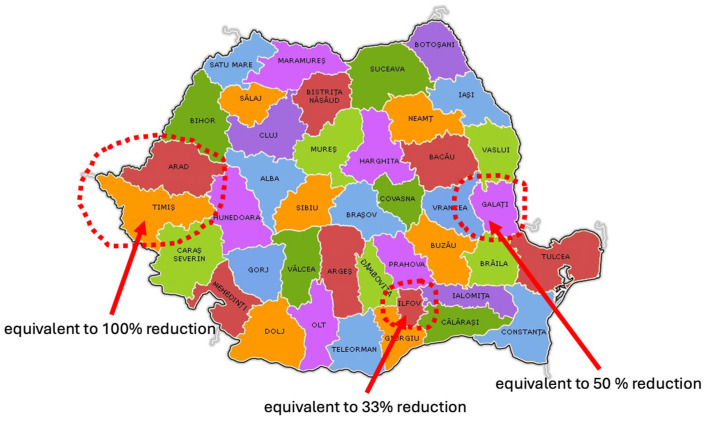
Equivalent utilized agricultural area (UAA) by counties in Romania for land use-reduction scenarios applied to the entire adult population. Source: Original by authors.

With the reduction of 100%, the largest decrease is observed, equaling a total of 11,131,127 ha, reducing land use by 733,898 ha compared to the 50% scenario and by a total of 1,067,443 ha compared to the baseline. This represents almost the cumulative UAA of two large-sized counties in Romania, Arad and Timis (1,113,352.75 ha) [49] (Figure 3).

It is emphasized that the gradual reduction in the consumption of animal products leads to a significant decrease in the need for agricultural land, with a direct impact on the conservation of natural resources and the reduction of the ecological footprint. Reducing 1,067,443 hectares in the use of agricultural land by fully switching to a plant-based diet is not just a statistical victory, it’s a statement of possibility. It shows that through informed and courageous choices in food policy and our personal preferences, we can contribute to a greener landscape and a healthier future [49,50,51].

## 4. Discussion

**Figure 4:**
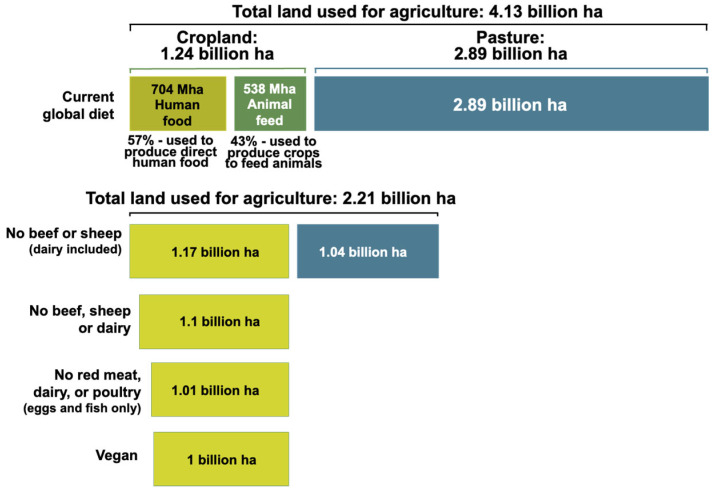
Global agricultural land use distribution for food production. Source: Original adapted by authors after [15].

The global distribution of agricultural land use for food production highlights a significant imbalance between the areas dedicated to food for human consumption and those used for animal feed [52,53]. Of the total 2.89 billion hectares of agricultural land, only 704 million hectares are directly allocated to human food, while 538 million hectares are dedicated to animal feed production. This reality highlights the inefficiency of animal-based food systems, which require much larger amounts of resources for a relatively small contribution to global caloric intake. Adopting plant-based diets can address this inefficiency, offering significant opportunities to save land and reduce the impact on ecosystems in the context of a growing global population and the urgent need for sustainable solutions [15] (Figure 4).

Referring to the total impact of replacing animal proteins with plant-based proteins, one of the major aspects highlighted by the results is the significant potential for saving agricultural land when animal protein is gradually replaced by plant sources. From savings of 84,020 hectares in Scenario 1 (33%) to 333,546 hectares in Scenario 2 (50%) and finally a substantial reduction of 1,067,443 hectares in Scenario 3 (100%), the results demonstrate that major improvement of land sustainability is possible by changing eating habits and moving to a plant-based diet.

Interpretation of this reduction shows that as the replacement rate of animal protein increases, land savings become more and more apparent. Although a full change (100%) generates the greatest impact, even a modest 33% reduction can have considerable benefits for reducing land pressure.

In terms of the efficiency of the different categories of food of animal origin, meat categories with the greatest land use impact, such as beef and dairy, remain problematic even after 33% or 50% replacements. For example, in the case of beef, land use is reduced by only 9.69 m² in Scenario 2, which underlines the inefficiency of this protein source from an ecological perspective. Also, dairy products, while generating significant savings in the intermediate replacement scenarios, continue to have a massive land footprint.

This finding suggests that to achieve real sustainability benefits, reducing the consumption of high-impact products such as beef and dairy products is essential. In contrast, poultry and fish proteins show considerable savings whether their consumption is partially or totally replaced by vegetable protein, indicating greater land use efficiency.

It is important to note that all our scenarios involve substitutions with a specific combination of plant sources (50% soy, 30% peas, and 20% potato), but this combination can be changed and adapted to both consumer preferences and the usage needs of the land. Romanians have a high preference for potatoes, which require almost as much land for 100 g of protein as eggs (potatoes 5.18 m²/100 g protein, eggs 5.65 m²/100 g protein), but potatoes can be replaced by corn, which need only 3.09 m²/100 g protein, or with wheat or rye, which require only 3.16 m²/100 g protein. This adjustment in the choice of replacement source can not only improve land use efficiency but also better align the transition with local dietary preferences and long-term SDGs.

Looking at the benefits of plant-based diets, research results confirm that a diet based on plant-based proteins, either partial (33%, 50%) or total (100%), can help reduce pressure on agricultural land, which allows for more efficient resource management. Soybeans, peas, and potatoes, the plant sources selected to replace animal proteins, are shown to be effective in saving land, especially compared to animal products such as beef, pork, and dairy products. This finding is particularly important in the context of climate change and global population growth, where the sustainability of food systems plays a crucial role in reducing the ecological footprint [54,55].

These results are particularly for Romania, a country with a strong tradition in the consumption of red meat and dairy products, reconfirmed over time, independent of socio-political conditions [56,57]. The proposed dietary changes, even partial ones, could significantly contribute to reducing the pressure on agricultural land, thereby freeing up space for other uses, such as reforestation or biodiversity protection.

The transition to more sustainable diets in Romania, although it may seem challenging from a cultural and economic point of view, could bring significant benefits not only for the environment but also for the health of the population. Reducing the consumption of animal protein, especially high-impact products such as beef, could help achieve national and international goals related to sustainability and resource management [58,59].

Assessing the challenges of transition scenarios, each of the three scenarios analyzed demonstrates that a gradual transition can have clear benefits. However, the complete replacement (100%) of animal proteins with plant-based proteins, although it generates the greatest land savings, could encounter difficulties in adoption, given the traditional dependence of Romanians on animal products. Therefore, intermediate scenarios, such as 33% or 50% replacement, may be more achievable and may constitute a first step towards wider change.

The limitations and shortcomings of the research, which should be covered by future research directions, are based on the fact that, in practice, the decrease is not proportional to the reduction scenarios, indicating the complexity of the production system and possible limitations in the complete substitution of animal products with plant alternatives. There are multiple reasons for this phenomenon:

- *Different efficiencies of plant sources.* The replacement of animal protein with vegetable protein is not linear in terms of land use. For example, soybeans have a much smaller land use than peas or potatoes, and the substitution is made with a mix of plant products, not with a single source [22,46]. Thus, depending on the plant sources selected for replacement, the decrease in land use may not be directly proportional to the percentage reduction in consumption of animal products.
- *Differences in land use intensity between animal products.* For example, beef has a much greater impact on land use than chicken or eggs [22,46]. Therefore, reducing beef consumption will have a greater impact on saving land than reducing egg consumption. If a small percentage of beef is replaced, it will generate a relatively greater decrease in land use than a similar amount of eggs or fish.
- *Local and specific factors for Romania.* Agricultural conditions in Romania, including farming practices and types of crops grown, can influence land use efficiency [6,7]. For example, some crops may have lower yields due to local soil or climate, which limits total land reduction even when replacing animal protein.

In the context of Romania, recent research on the impact of diets on the use of agricultural land has highlighted the need to change dietary habits in order to reduce the national ecological footprint [14]. Romania, a country with a strong agricultural tradition, uses a large part of its agricultural area to raise animals and grow the food they need. Globally, data show that around 83% of agricultural land is dedicated to livestock, while animal products contribute only 18% of global caloric intake. The same trend can be observed in Romania, where the high consumption of meat, especially pork and beef, has a significant impact on the environment [14,54].

Animal products such as beef and lamb are much more resource-intensive, including their use of agricultural land, compared to vegetable foods. For example, to produce 100 g of beef protein, about 70 times more land is needed than for the same amount of soy protein. This highlights the inefficiency of animal protein production, both globally and nationally [16,18].

Adopting a plant-based diet would have a significant impact on agricultural land use in Romania. If the population switched to a plant-based diet, the use of agricultural land could decrease considerably, helping to free up vast areas of land that could be used for reforestation or other environmentally beneficial activities. This would also help combat biodiversity loss and restore damaged natural ecosystems [14,21].

Crops such as cereals and legumes are excellent sources of protein and energy and are much more land-use efficient than animal products. Wheat and rice, for example, have a significantly smaller ecological footprint than beef or lamb, and legumes such as peas and soy provide quality protein with minimal environmental impact [15].

In Romania, technological progress in agriculture led to increased crop yields, which allowed the reduction of land needed for food production. On the other hand, advances in the livestock sector have been more limited, maintaining inefficiencies in the use of land for animal husbandry [20,29]. Reducing meat consumption, especially beef and lamb, in favor of a plant-based diet could make a significant contribution to reducing Romania’s ecological footprint, at the same time supporting national and international environmental protection objectives and combating climate change.

At the same time, it is very important to highlight the aspects related to the health of the Romanian population, regarding the consumption of proteins of animal origin. In Romania, the prevalence of cardiovascular diseases and nutritional problems, such as diabetes and obesity, has increased significantly in recent decades, affecting more and more adults. One of the main factors contributing to this alarming trend is a diet high in animal products, especially red and processed meat, high-fat dairy products, and ultra-processed foods [60,61].

**Figure 5:**
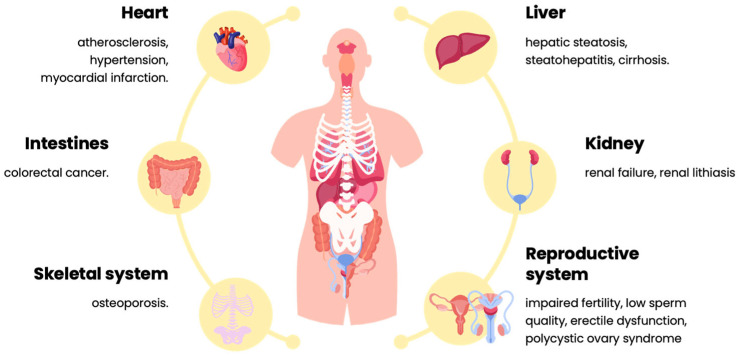
Health impacts associated with the consumption of animal-based proteins. Source: Original by authors.

Studies show that diets high in saturated fat and cholesterol, mainly from beef, pork, and dairy products, are strongly linked to the development of cardiovascular disease. Excessive consumption of such products leads to an increase in the level of LDL (“bad”) cholesterol and, implicitly, to a higher risk of hypertension, atherosclerosis, and heart attack [62,63] (Figure 5). In addition, these diets contribute significantly to obesity, an increasingly widespread problem in Romania, which, in turn, is a major risk factor for type 2 diabetes.

In contrast, plant-based foods such as legumes, whole grains, fruits, and vegetables have demonstrated significant health benefits. They are rich in fiber, vitamins, and antioxidants, and plant-based diets are associated with a reduced risk of chronic diseases, including cardiovascular disease and diabetes. Eating more plant-based foods can improve lipid profiles and help manage body weight, thereby reducing the risk of many diet-related conditions.

Statistical data suggest that in Romania, a large part of the adult population suffers from cardiovascular diseases, and type 2 diabetes is becoming more and more widespread. In addition, obesity rates are increasing, especially among middle-aged adults. These health problems can be directly correlated with a diet rich in animal products and poor in plant foods, thus contributing to the burden of chronic diseases in Romania.

By adopting a plant-based diet, the positive impact would not only be on the environment, but also on public health. Reducing the consumption of red and processed meat in favor of plant-based proteins could help reduce the incidence of cardiovascular diseases, obesity, and diabetes, thus offering a viable solution for improving the health of the Romanian population. This dietary change should become a priority for both the population and the health authorities in order to reduce the incidence of chronic diseases and improve the quality of life.

## 5. Conclusions

Dietary changes have become a central theme in sustainability debates, particularly in the context of agriculture and natural resource use. The present study explores the potential of a plant-based diet in Romania, demonstrating that a transition to plant-based protein sources can have a significant impact on agricultural land use. In a country with a strong agricultural tradition and a food culture based on animal products, these scenarios of replacing animal proteins with plant proteins open a viable perspective for optimizing resources and reducing pressure on land.

The results of our research highlight that by reducing the consumption of proteins of animal origin, even partially, Romania could save considerable areas of agricultural land. The analyzed scenarios with 33%, 50%, and 100% replacements show a clear trend of land-saving, with the highest benefits in the full replacement scenario. For example, in the scenario of 100% replacement of animal protein with plant-origin sources, land use could be reduced from 0.816 ha per capita up to 0.745 ha per capita and a cumulative reduction of 1,067,443 ha of land. This reduction could generate significant benefits not only for the protection of agricultural resources but also for the conservation of biodiversity and the fight against climate change.

In addition, this study shows that different types of animal feed have varying impacts on land use. Beef and dairy continue to dominate land use, even in partial reduction scenarios. These product categories have a much larger ecological footprint compared to other types of meat or plant-based products, suggesting that a significant reduction in beef and dairy consumption is necessary to maximize land savings.

The study emphasizes the importance of integrating a more sustainable diet into the eating habits of Romanians, both to protect natural resources such as water, land and to improve biodiversity and public health. In Romania, the prevalence of cardiovascular diseases and nutritional problems such as diabetes and obesity has increased significantly in recent decades, largely due to a high consumption of animal products [64,65]. A plant-based diet rich in fiber and antioxidants can help reduce health risks, providing a viable solution to reducing the incidence of chronic disease and improving quality of life.

This research provides solid evidence that adopting a plant-based diet in Romania could bring major benefits in terms of agricultural land use, environmental protection, and population health. The transition to such a diet is not only an ecological option but also a necessity in the context of global challenges related to natural resources and public health [59,60]. Public policies should support this change through education and awareness initiatives, promoting a sustainable and nutritionally balanced lifestyle.

It is important to emphasize the contribution that the transition to a plant-based diet can provide to the achievement of Sustainable Development Goal 12 (SDG 12), which aims to ensure sustainable patterns of consumption and production. SDG 12 aims to reduce the negative impact of human activities on the environment by promoting responsible and efficient consumption of natural resources, as well as by reducing food waste and emissions generated by the food chain [64,65].

In Romania, where a significant part of the agricultural land is devoted to meat and dairy production, switching to a plant-based diet offers multiple benefits. Reducing the consumption of animal products could reduce intensive land use, water consumption, and greenhouse gas emissions while contributing to the conservation of ecosystems and biodiversity [20,29].

In addition, this dietary shift supports global goals, such as improving health (SDG 3) and combating climate change (SDG 13), by decreasing the prevalence of chronic diseases and reducing emissions associated with food production [20,29,64,65].

Achieving SDG 12 requires a reassessment of how we consume and produce food. Our research demonstrates that a significant dietary change in Romania, from an animal-based diet to a plant-based one, would bring substantial benefits not only in terms of sustainable agricultural land use but also for public health and environmental protection.

This change does not just mean reduced agricultural land; it means restored habitat for wildlife, less carbon emissions from more extensive agriculture, and a better balance between our needs and those of the planet we share [66,67,68]. Behind every hectare of land saved are hundreds of species of plants and animals that benefit from a healthier environment, and every step towards reducing the use of agricultural land is a step towards ensuring that future generations inherit a world where the diversity of life continues to thrive.

Every decision we make, from policies to personal consumption choices, shapes this land use map. In the face of global ecological challenges, we are invited to be the heroes of our own future, to make choices that write not just numbers on a graph but the story of a sustainable future.

## Figures and Tables

**Table 1:** Protein content of food of animal origin [22].

Entity	Poultry	Boneless Beef	Sheep & Goat	Pig	Meat Other *	Fish & Seafood	Eggs	Milk
Protein g/100 g	17.1	18.5	14	11	16.1	10.9	10.7	3.3

* turkey, rabbit.

**Table 2:** Annual per capita consumption of animal products in Romania [23].

Meat and Fish & Seafood kg/Year/Capita						Eggs kg/Year/Capita	Milk kg/Year/Capita
75.24 of Which:							
Poultry	Beef	Sheep & goat	Pig meat	Meat other	Fish & seafood	12.84	599.32
22.21	5.05	2.8	36.9	0.19	8.09		

**Table 3:** Land use for 33% replacement of animal protein with plant-based protein sources.

Specification	C (kg)	P (%)	*LU_animal_* (m^2^)	*LU_initial_* (m^2^)	Replaced P (kg)	*LU_replaced_* (m^2^)	*LU_final_* (m^2^)
Poultry	22.21	17.1	7.06	267.96	12.53	39.40	228.55
Beef	5.05	18.5	163.60	1528.39	3.08	9.69	1518.70
Sheep and goat	2.8	14	184.81	724.47	1.29	4.07	720.40
Pig meat	36.9	11	10.73	435.50	13.39	42.11	393.39
Meat other	0.19	16.1	7.06	2.16	0.10	0.32	1.84
Fish and seafood	8.09	10.9	3.69	32.52	2.91	9.15	23.37
Eggs	12.84	10.7	5.65	77.63	4.53	14.25	63.38
Milk	599.32	3.3	27.12	5363.91	65.27	205.20	5158.72
TOTAL m^2^				8164.59			8108.35
TOTAL ha				0.816			0.811

Note: For definitions of C, P, and LU, please refer to Section 2. Materials and Methods, Section 2.2, Applied Formulas.

**Table 4:** Land use for 50% replacement of animal protein with plant-based protein sources.

Specification	C (kg)	P (%)	*LU_animal_* (m^2^)	*LU_initial_* (m^2^)	Replaced P (kg)	*LU_replaced_* (m^2^)	*LU_final_* (m^2^)
Poultry	22.21	17.1	7.06	267.96	18.99	59.70	208.26
Beef	5.05	18.5	163.60	1528.39	4.67	14.69	1513.71
Sheep and goat	2.8	14	184.81	724.47	1.96	6.16	718.30
Pig meat	36.9	11	10.73	435.50	20.30	63.81	371.69
Meat other	0.19	16.1	7.06	2.16	0.15	0.48	1.68
Fish and seafood	8.09	10.9	3.69	32.52	4.41	13.86	18.66
Eggs	12.84	10.7	5.65	77.63	6.87	21.60	56.04
Milk	599.32	3.3	27.12	5363.91	98.89	310.90	5053.01
TOTAL m^2^				8164.59			7941.34
TOTAL ha				0.816			0.794

Note: For definitions of C, P, and LU, please refer to Section 2. Materials and Methods, Section 2.2, Applied Formulas.

**Table 5:** Land use for 100% replacement of animal protein with plant-based protein sources.

Specification	C (kg)	P (%)	*LU_animal_* (m^2^)	*LU_initial_* (m^2^)	Replaced P (kg)	*LU_replaced_* (m^2^)	*LU_final_* (m^2^)
Poultry	22.21	17.1	7.06	267.96	37.98	119.41	148.55
Beef	5.05	18.5	163.60	1528.39	9.34	29.37	1499.02
Sheep and goat	2.8	14	184.81	724.47	3.92	12.32	712.14
Pig meat	36.9	11	10.73	435.50	40.59	127.61	307.89
Meat other	0.19	16.1	7.06	2.16	0.31	0.96	1.20
Fish and seafood	8.09	10.9	3.69	32.52	8.82	27.72	4.80
Eggs	12.84	10.7	5.65	77.63	13.74	43.19	34.44
Milk	599.32	3.3	27.12	5363.91	197.78	621.81	4742.11
TOTAL m^2^				8164.59			7450.14
TOTAL ha				0.816			0.745

Note: For definitions of C, P, and LU, please refer to Section 2. Materials and Methods, Section 2.2, Applied Formulas.

**Table 6:** Resident population in Romania [48].

Category	Total Resident Population	Resident Population %
0–18 y.o.	4,112,985	21.59
18–>85 y.o.	14,940,830	78.41
Total	19,053,815	100

## Data Availability

Data were obtained from the National Institute of Statistics Romania and are available at https://www.recensamantromania.ro/rezultate-rpl-2021/rezultate-definitive/ and https://insse.ro/cms/sites/default/files/field/publicatii/anuarul_statistic_al_romaniei_carte_ed_2023-ro.pdf with open access to the National Institute of Statistics Romania (https://insse.ro/cms/en/content/information-services-0, all accessed on 1 September 2024).
